# Stability of methylnicotinate in aqueous solution as utilized in the 'niacin patch test'

**DOI:** 10.1186/1756-0500-1-89

**Published:** 2008-09-24

**Authors:** Brian M Ross, Martin Katzman

**Affiliations:** 1 Northern Ontario School of Medicine, Lakehead University, Thunder Bay, Ontario, Canada; 2 The START Mood and Anxiety Disorders Clinic, Toronto, Ontario, Canada

## Abstract

**Background:**

The topical application of methylnicotinate results in a localized vasodilatatory response which has been found to differ from that observed to occur in healthy controls in a variety of medical conditions. The stability of the drug in aqueous solution is unclear while difficulties can be encountered when preparing methylnicotinate solutions for this purpose. To aid in the determination of how long solutions of the drug should be stored before discarding we have used a collection of aged batches of methylnicotinate to determine the stability of the drug in aqueous solution.

**Findings:**

The degradation of methylnicotinate was determined in batches which had been stored at 4°C for between 5 and 1062 days prior to analysis by High Performance Liquid Chromatography. The major degradation product of methylnicotinate was nicotinic acid which formed at an approximate rate of 0.5% of the starting methylnicotinate concentration per annum. Furthermore, the ability of methylnicotinate solutions of different ages to induce vasodilatation was assessed in healthy volunteers. No significant difference in vasodilatatory response was apparent between batches which had been stored for between zero and 1057 days.

**Conclusion:**

Methylnicotinate exhibits excellent chemical and biological stability in solution facilitating its use in clinical applications.

## Background

Systemic administration of the B vitamin niacin (nicotinic acid) produces a generalized vasodilatatory response [1]. This occurs due to nicotinic acid stimulating the production of prostaglandin D_2 _from arachidonic acid in the skin which subsequently acts upon capillary endothelial cells to cause vasodilatation [1,2]. The methylester of nicotinate, methylnicotinate, has a similar vasodilatatory effect, however the ester can be applied transcutaneously resulting in a localized, rather than generalized, response [3]. Methylnicotinate has found application as a rubefacient [4] and in the development and testing of anti-inflammatory drugs [5]. Topical application of methylnicotinate has also been used to investigate the inflammatory response in a variety of disorders including autism [6], Huntington's disease [7], social phobia [8], major depressive disorder [9,10] and schizophrenia [3,10-18]. In this latter application varying doses of methynicotinate are applied to the skin (normally the forearm) in the form of a patch (frequently, if inaccurately, referred to as a 'niacin patch'). The resulting vasodilatatory response is then measured either by the visual estimation of erythema [4,9] or measuring superficial blood flow by laser Doppler spectrometry [8,13,14]. Such studies have been complicated by the fact that the stability of methylnicotinate in aqueous solution is unknown, necessitating the preparation of new solutions on a regular basis. Besides introducing an additional source of error, the production of new batches of the drug is made difficult due to the high degree of hygroscopicity of solid methylnicotinate. This results in storage and handling difficulties and an inevitable need to regularly discard batches of the solid ester. It would therefore be beneficial to store the aqueous solution for extended periods of time. The stability of methylnicotinate in aqueous solution is not known however, and hence it is unclear for how long the solutions can be stored for. As such many batches of the aqueous drug have been prepared and stored over several years. This has allowed us to investigate the stability of methylnicotinate in solution as well as testing the effect of storage time on the ability of aged preparations to induce erythema, as an aid to determining the storage life of the solution.

## Analysis of methylnicotinate degradation in aqueous solution

Methylnicotinate (NHS Highland Labs, UK) was prepared at a concentration of 1 M in water and stored at 4°C in glass containers for between 5 – 1062 days prior to analysis. All batches were prepared using an identical protocol in the same laboratory. No differences in colour or clarity were discernable by visual inspections between batches of different ages, and no batches contained any visible precipitate. The chemical stability of methylnicotinate was investigated using High Performance Liquid Chromatography (HPLC). To accomplish this 1 M stock solutions of methylnicotinate were diluted and methylnicotinate and a degradation product, nicotinic acid, determined in quadruplicate by HPLC using the following analysis parameters: injection volume: 20 μL; separator column: Supelco (Sigma, UK) supelcosil TM LC-18 ODS-18, (length: 25 cm, internal diameter: 4.6 mm, particule size: 5 μm); eluant: 25% v/v methanol in water; eluant flow rate: 1.5 mL/min. Eluted compounds were detected by UV @ 263 nm using an Applied Biosystems Spectroflow 757 UV-vis detector and quantified by comparision with nicotinic acid and 6-methylnicotinate standards (Aldrich, UK). Nicotinic acid had a retention time of 1.4 minutes and a limit of detection of 0.04 μg/ml, while methylnicotinate exhibited a retention time of 7.3 minutes and a limit of detection of 0.05 μg/ml. Within-day variance expressed as the standard deviation as a percentage of the mean was 0.8%, and between day variance was 1.5%. The detection of both nicotinate and methylnicotinate showed a linear response with respect to concentration (Pearson correlation coefficient of detector response vs. applied quantity was 0.98) over the range tested using freshly prepared standards (maximum concentration tested was 150 μg/ml).

**Figure 1:**
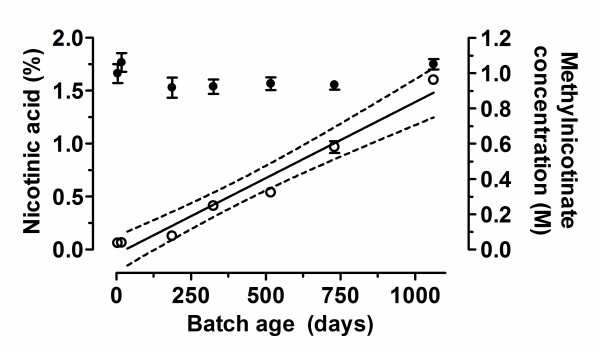
**Relationship between batch age, methylnicotinate concentration and relative nicotinic acid content of 1 M aqueous solutions of methylnicotinate**. Batches of 1 M methylnicotinate were stored at 4°C for increasing lengths of time prior to analysis by HPLC. Filled circles, plotted on the right axis, show the measured concentration of methylnicotinate in each batch. Open circles, plotted on the left axis, show the percentage of nicotinic acid relative to the total methylnicotinate plus nicotinic acid concentration in each batch. Values shown are the mean of quadruplicate determinations; bars indicate the standard deviation. The 'best-fit' linear regression line for the correlation between batch age and nicotinic acid content is indicated by a solid line; the dotted lines indicate the 95% confidence intervals for this line based on the available data.

**Figure 2:**
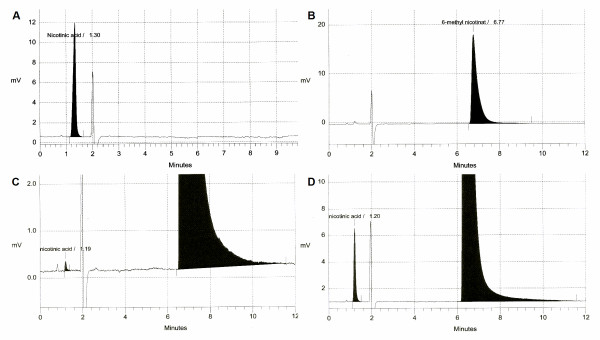
**Analysis of methylnicotinate solutions by HPLC**. Representative chromatograms are shown for (A) nicotinic acid standard, (B) 6-methyl nicotinate standard, (C) 6-methylnicotinate solution stored for 5 days at 4°C prior to analysis and (D) 6-methylnicotinate solution stored for 1062 days at 4°C prior to analysis.

The concentration of methylnicotinate in each batch did not differ significantly between batches of different ages, all having the expected measured concentration of approximately 1 M (Figure 1), and there was no significant linear relationship with batch age (Pearson correlation coefficient was 0.001; *P *> 0.05). Qualitative assessment of the chromatograms of freshly prepared methylnicotinate indicated that apart from the compound itself, the predominant contaminant co-eluted with nicotinic acid. Representative chromatograms are shown in Figure 2. The concentration of nicotinic acid in a batch of methylnicotinate prepared 5 days previously was 0.060 ± 0.002% (mean ± SD nicotinate as a percentage of methylnicotinate analysed in quadruplicate). As illustrated in Figure 1 the degradation of methylnicotinate to form nicotinic acid proceeded linearly with respect to time (Pearson correlation coefficient = 0.98) at a rate of 0.00150% per day (95% confidence interval was 0.001270% to 0.00172%) or 0.54% per 365 day year (95% confidence interval was 0.47% to 0.63%).

## Erythema response

**Figure 3:**
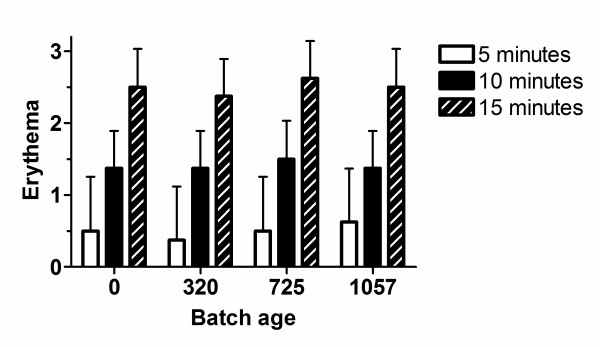
**Lack of effect of storage time upon extent of erythema induced by methylnicotinate**. 10 mM aqueous solutions of methylnicotinate prepared from stock solutions which had been stored for various lengths of time at 4°C were applied to the forearm of 8 healthy volunteers and the induced erythema assessed visually at 5, 10 and 15 minutes post-application using a 4-point scale. Values shown are mean erythema; bars indicate the standard deviation.

In addition to the HPLC analysis, the vasodilatatory activity of aged solutions was also investigated. The four batches utilised had been stored for 0, 320, 725 and 1057 days respectively at the time of testing. The 1 M stock solutions were diluted to 10 mM and 1 cm^2 ^paper patches were impregnated with approximately 30 μl of the solution as previously described in detail [4]. The patches were applied to the forearms of eight healthy volunteers (4 male, 4 female, age was 44 ± 12 years [mean ± SD]. The area of skin to which the patch was applied was examined for, and was free of, damage. The skin patch procedure had been reviewed and approved by NHS Highlands Research Ethics Committee. All subjects gave informed consent including that the data obtained from their participation may be published in aggregate format. Erythema, monitored at 5, 10 and 15 minutes after application, was assessed visually using a 4 point scale (0 – no erythema, 1 – perceptible but slight erythema, 2 – moderate erythema, 3 – pronounced erythema). As illustrated in Figure 3, the batch age had no effect upon the extent of erythema (2-way ANOVA with factors time and batch age). There was no statistically significant interaction between time and batch age (*F*_6,56 _= 0.21; *P *> 0.9) nor was their a significant effect of batch age (*F*_3,56 _= 0.16; *P *> 0.9), although there was a statistically significant effect of time (*F*_2,56 _= 175.34; *P *< 0.0001)). The testing of the ability of each batch of methylnicotinate to induce erythema was performed using a single dose. We cannot rule out that an effect of batch age would have been observed using different, especially lower, concentrations, although we consider such a possibility unlikely. It has been reported that methylnicotinate-induced erythema is reduced with age [19]. We did not observe, however, a statistically significant correlation between age and erythema (for example the Pearson correlation coefficient between erythema at 15 minutes and subject age was – 0.31; *P *< 0.05), although the small sample size may have prevented us from detecting such a relationship.

## Conclusion

Our data suggests that the degradation of methylnicotinate proceeds slowly in aqueous solution. Chromatographic analysis indicates that the methylester gradually hydrolyses at the rate of approximately 0.5% per annum to form nicotinic acid and, presumably, methanol. No other degradation products were noted. This rate of degradation had no apparent effect upon the ability of aged batches, prepared up to approximately 3 years prior, to induce erythema in healthy subjects. Since erythema was determined visually it remains a possibility that detection methods which are able to detect smaller changes in blood flow, such as laser Doppler spectroscopy, would have detected an effect of storage time. Such a possibility is unlikely, however, given that the extent of degradation in batches prepared 1062 days prior (approximately 1.5%) is rather lower than the test-retest variance of laser Doppler measurements which are in the range of 5 – 10% (BMR, unpublished observations). It is important to note that the methylnicotinate solutions used in this study had a concentration of 1 M and had been stored refrigerated at 4°C in glass containers. It is possible that storage of solutions at different concentrations, or in containers made from substances other than glass would have given contrary results due to, for example, absorption to the surface of the container. Moreover, storage at higher temperatures e.g. room temperature would likely have resulted in higher rates of degradation, although it is common for test reagents to be stored in a refrigerator. Nevertheless, our overall conclusion is that methylnicotinate solutions can be stored for extended periods of time at 4°C in glass containers without the occurrence of extensive chemical degradation or loss of biological activity. This finding may aid the use of methylnicotinate for research purposes or for future clinical diagnostic uses.

## Competing interests

The authors declare that they have no competing interests.

## Authors' contributions

BMR carried out the chemical analysis and erythema study, while both BMR and MK analysed the data and wrote the manuscript. Both authors approved of the final version of the manuscript.
